# Physicochemical, Textural, and Antioxidant Attributes of Yogurts Supplemented with Black Chokeberry: Fruit, Juice, and Pomace

**DOI:** 10.3390/foods13203231

**Published:** 2024-10-11

**Authors:** Sergiu Pădureţ, Cristina Ghinea, Ancuta Elena Prisacaru, Ana Leahu

**Affiliations:** Faculty of Food Engineering, Stefan cel Mare University of Suceava, 720229 Suceava, Romania; sergiu.paduret@fia.usv.ro (S.P.); ancuta.prisacaru@fia.usv.ro (A.E.P.); analeahu@fia.usv.ro (A.L.)

**Keywords:** chokeberry, physicochemical analysis, pomace, texture, total phenolic content, yogurt

## Abstract

The fruit, juice, and pomace of black chokeberry (*Aronia melanocarpa*) are a rich source of phenolic compounds and can be used to obtain enriched dairy products. Chokeberry fruit, due to its astringent taste, is less favorable or even unacceptable to consumers and is usually processed into juice, resulting in large quantities of pomace, which is often discarded as waste. The aim of this study is to valorize chokeberry fruit, juice, and pomace by incorporating them in different percentages (1, 2, and 3%) into yogurt as functional ingredients. The physicochemical (total solids content, fat, protein, titratable acidity, pH, color), textural (hardness, adhesion, cohesiveness, springiness, gumminess, and chewiness), antioxidant (DPPH scavenging activity and total phenolic content), and sensory characteristics of supplemented yogurts were investigated. The results showed that the addition of chokeberry pomace in yogurt increased their total solids content (from 11.46 ± 0.18% for the plain yogurt sample to 13.71 ± 0.18% for the yogurt sample with 3% chokeberry pomace), while the addition of fruit and juice decreased the protein content of the yogurt samples (from 4.35 ± 0.11% for the plain yogurt sample to 3.69 ± 0.15% for the yogurt sample with 3% chokeberry fruit and to 3.84 ± 0.1% for the yogurt sample with 3% chokeberry juice). There was no statistically significant change in the fat content of all samples of chokeberry-supplemented yogurt compared to plain yogurt. The pH of the yogurt samples decreased with the increase in the percentage of chokeberry fruit, juice, and pomace added to the yogurt (from 4.50 for the plain yogurt samples to 4.35, 4.30, and 4.20 for the yogurt samples supplemented with 1, 2, and 3% black chokeberry pomace). Inhibition of DPPH radical formation was higher in the yogurt samples with chokeberry fruit (57.84 ± 0.05%, 73.57 ± 0.11%, and 75.38 ± 0.05% inhibition for the samples with 1, 2, and 3% fruit) and pomace (up to 64.8 ± 0.11% inhibition for the sample with 3%), while total phenolic content decreased (from 392.14 ± 2.06 to 104.45 ± 2.63 µg/g) as follows: yogurt with chokeberry pomace > yogurt with chokeberry fruit > yogurt with chokeberry juice. The yogurt samples with the highest acceptance scores were the samples with 3% and 2% black chokeberry fruit, while the lowest acceptance score was obtained for the yogurt sample with 3% black chokeberry pomace. Chokeberry fruit, juice, and pomace can improve the physicochemical, textural, and antioxidant characteristics of yogurt, emphasizing that the antioxidant effect of yogurt could be substantially improved by the addition of chokeberry pomace due to its high phenolic content, while incorporation into yogurt is another way to valorize this by-product.

## 1. Introduction

Worldwide, the popularity of black chokeberry (*Aronia melanocarpa*) has increased with the growing interest in healthy foods [1]. Originally from North America (where it was commonly used to treat colds, coughs, and fevers [2]), black chokeberry was brought to Europe (in the 20th century) and nowadays is very popular in northern, central, and eastern Europe [3,4,5]. Since 1986, chokeberry has also been cultivated in Romania according to Dinu et al. [3], and has been adapted to the climatic and soil conditions of the country [6]. Black chokeberry fruit contains polyphenols like proanthocyanidins (4790 mg/100 g fresh weight), anthocyanins (mainly cyanidin-3-O-galactoside and cyanidin-3-O-arabinoside), quercetin (1.5–71.0 mg/100 g fresh weight), and phenolic acids (chlorogenic acid and neochlorogenic acid) [7,8], as well as pectin and fat in low amounts, and vitamins and minerals [9]. The beneficial compounds like polyphenols in black chokeberry (from 2000 mg/100 g to 8000 mg/100 g in dry weight) are useful in the treatment of diabetes, cardiovascular disease, hypercholesterolemia, hyperlipidemia, and hypertension [2,10]. Nevertheless, the high quantity of polyphenols (especially procyanidins [11]) gives black chokeberry an astringent taste, which makes the unprocessed fruit less favorable or even unacceptable to consumers [7,12]. According to D’Alessandro et al. [13], the production of juices, jams, and wines from black chokeberry is more popular in European countries, with these foods being more easily accepted by consumers. However, chokeberry juice is also astringent with moderate sugar content and low pH [12]. Also, large amounts of chokeberry pomace (10% pulp, 28–35% skin, and 60–70% seeds) result after juice processing [5,13], which is frequently discarded as waste causing environmental problems [12]. Rich in anthocyanins and total dietary fiber, chokeberry pomace can be valorized by using it in food products (bread [14], beef patties [15], ready-to-eat extruded cereals [16], and yogurt [17], which is a highly appreciated worldwide product [18]), thus reducing the amount of waste generated [19]. The addition of berries, juice, and pomace to various food products has increased in recent years and it seems that the key factors for consumer acceptance of food products are flavoring and more recently enrichment with a variety of bioactive compounds [20]. The use of fruit pomace in various food products can contribute to the circular economy, while the addition of natural antioxidant sources satisfies consumer demand for “clean label” food [21]. In the dairy industry, the high nutritional value and bioactive content of yogurt are increased by the addition of fruit and fruit juices [22,23]. Although the addition of chokeberry fruit [24], chokeberry juice [22,25], and chokeberry pomace [17] in yogurt samples has already been the target of investigations, there are no studies providing a complete comparison of the effect of chokeberry fruit, juice, and pomace on physicochemical, textural, and antioxidant attributes of yogurt. Therefore, chokeberry fruit, juice, and pomace in different amounts (0, 1, 2, and 3%) were added to the yogurt formulations. The effects of black chokeberry (fruit, juice, and pomace) on the total solids, fat, protein, titrable acidity, pH, color parameters (L*, a *, b*, C, YI, WI, h°), hardness (H), viscosity (V), adhesion (A), cohesiveness (Co), springiness (S), gumminess (G), chewiness (Ch), antioxidant activity, and sensory characteristics of the product were investigated. In this study, the main purpose was to investigate the potential of chokeberry pomace, compared to chokeberry fruit and juice, as an innovative and sustainable ingredient in the dairy sector by improving the nutritional profile of yogurts and reducing waste, thus supporting the implementation of a circular economy model for environmental conservation.

## 2. Materials and Methods

### 2.1. Milk, Starter Culture, Black Chokeberry: Fruit, Juice, and Pomace

**Figure 1:**
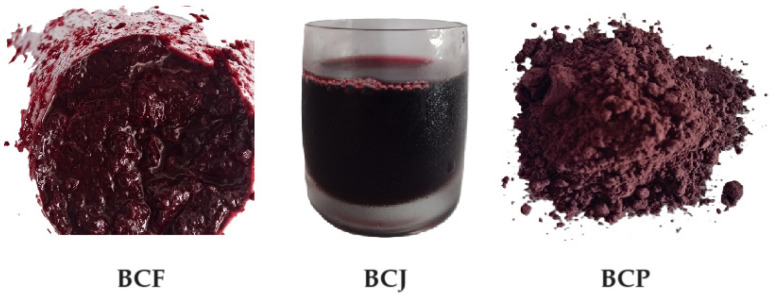
The black chokeberry fruit puree (BCF), black chokeberry juice (BCJ), and black chokeberry pomace (BCP) used to supplement yogurt.

Commercial pasteurized and homogenized cow’s milk (3.5% fats, 4.5% carbohydrates, and 3% proteins) was purchased from a local market in Suceava, Romania. Black chokeberry fruit with 11.2% carbohydrates, 1.5% proteins, 0.4% fats, and 1.3% fibers; black chokeberry juice with 13% carbohydrates, 0.1% proteins, under 0.5% fats, and under 0.5% fibers; and black chokeberry pomace with 7.6% carbohydrates, 7.3% proteins, 4.1% fats, and 70% fibers were obtained from a local producer (Figure 1). According to the producer, the chokeberry fruit composition (protein, fat, carbohydrate, and total dietary fiber contents) was determined according to AOAC [26], while chokeberry pomace protein content was obtained by using the Kjeldahl method [27], fat content by using Soxhlet extraction [28], and total dietary fiber with the method described in [29]. Proximate analysis of chokeberry juice was performed by using AOAC [30]. Chokeberries have a high content of polyphenolic compounds with positive effects on human health (anti-inflammatory, immunomodulatory, and antimutagenic properties) due to their antioxidant activity. The total phenolic contents (TPCs) of chokeberry products were as follows: 1136 mg of GAE/100 g fruit, 408 mg of GAE/100 g juice, and 4332 mg of GAE/100 g pomace. These findings are in agreement with the literature data: Denev et al. [31] stated that the TPC ranged from 690 to 2556 mg of GAE/100 g of fresh weight and from 3440 to 7849 mg/100 g of dry weight. Nour [32] obtained 336.61 mg of GAE/100 g juice, while Konić Ristić et al. [33] indicated 586 mg of GAE/100 g juice, and Tolić et al. [34] determined 4233 mg of GAE/100 g chokeberry pomace. Lactic cultures (*Lactobacillus delbrueckii* subsp. *Bulgaricus* and *Streptococcus thermophilus*) for probiotic yogurt were purchased from Chr. Hansen (Selection medium 1, Chr. Hansen S.R.L. Brasov, Romania, 1 U/5 L milk).

### 2.2. Production of Yogurt Supplemented with Black Chokeberry

A total of 10 yogurt samples (Table 1) were prepared from pasteurized cow milk brought to 42 °C for the lactic culture (1 U/5 L milk) dosage and stored at this temperature until the pH reached a value of 4.5.

The black chokeberry fruit (BCF), black chokeberry juice (BCJ), and black chokeberry pomace (BCP) were added in different concentrations (1, 2, and 3%, Table 2) and thoroughly mixed, and then refrigerated at 4 °C for 24 h. A control sample (YCS) was produced using only pasteurized cow milk and lactic cultures.

**Figure 2:**
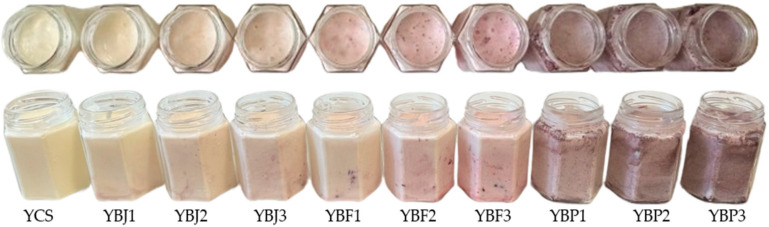
The yogurt samples supplemented with black chokeberry fruit and pomace.

The yogurt samples supplemented with black chokeberry fruit and pomace are illustrated in Figure 2.

### 2.3. Physicochemical Analysis

Determination of the total solids content of the yogurt samples was carried out at 102 °C ± 2 °C in a drying oven until the mass decreased by 1.0 mg or less, or increased between two successive weighings, according to ISO 13580 [35]. Before analysis, the yogurt samples were homogenized to facilitate the dispersion of the added products and brought to a temperature between 20 °C and 25 °C. Fat content was determined using the Gerber acid butyrometric method as described in the literature [36,37]. The total protein content in the yogurt samples was determined through the assessment of total nitrogen by applying the Kjeldahl method [38] using a Kjeldahl digestion unit (DK6, VELP Scientifica, Velate MB, Italy) and a Kjeldahl distillation–titration unit (UDK 127, VELP Scientifica, Velate MB, Italy), employing a nitrogen-to-protein conversion factor of 6.25 [39]. The results were expressed as a mass percentage. The titratable acidity was evaluated following the ISO11869 method [40] with an automatic titrator (Schott TITROLINE^®^ easy Titrator, SI Analytics GmbH, Mainz, Germany) using 0.1 M sodium hydroxide solution until the pH attained a value of 8.3. The pH was measured using a Mettler Toledo pH Meter (Mettler Toledo GmbH, Greifensee, Switzerland). The color evaluation of the yogurt samples was performed with a Konica Minolta CR-400 ChromaMeter (Konica Minolta, Tokyo, Japan) using a C illuminant and a 2° observer. For the brightness (L*), red (+a*), green (−a*), yellow (+b*), and blue (−b*) color parameters, the (CIE) L*a*b* (Commission Internationale de l’Eclairage) method was used [41]. From the measured color parameters, the tone (h^0^), chroma (C*), whiteness index (WI), and yellowness index (YI) were derived. The ChromaMeter calibration was made against a porcelain plate (Konica Minolta, Tokyo, Japan) [42].

### 2.4. Texture Analysis

The texture properties of the yogurt samples were evaluated by the Texture Profile Analysis method (TPA) with a Mark 10 ESM 301 texture meter (Mark 10 Corporation, Copiague, NY, USA), equipped with a 10 N load cell and a 5 cm diameter disc probe. The TPA was conducted at 150 mm/min until reaching a depth of 12 mm in the 30 mm yogurt column with a 70 mm diameter [43,44]. The MESUREgauge software version 2.1.1 (Mark 10 Corporation, Copiague, NY, USA) was used for the TPA curve registration at a speed reading of 12 points per second. From the load versus travel curves, hardness (H), viscosity (V), adhesion (A), cohesiveness (Co), springiness (S), gumminess (G), and chewiness (Ch) were the calculated texture parameters [45,46].

### 2.5. Antioxidant Activity

Extract preparation: A mass of 10 g of homogenized yogurt samples was mixed with 10 mL of 60% ethanol at room temperature, and after 30 min of stirring, the resulting mixture underwent centrifugation at 4000 rpm for 20 min using an OHAUS Frontier™ 5718R centrifuge (Switzerland). The obtained supernatant was stored in refrigeration conditions (4–6 °C) for subsequent evaluation of the antioxidant activity and total phenolic content [47].

Radical scavenging activity: The DPPH assay (2,2-Diphenyl-1-picrylhydrazyl) was utilized to evaluate the antioxidant potential of the yogurt samples, following the method outlined by El-Din et al. [48] with slight adjustments. A volume of 0.1 mL yogurt extract was combined with 2.9 mL of ethanolic DPPH solution of 0.1 mM [49]. The resulting mixture was vigorously vortexed for 1 min and then incubated in darkness at room temperature for 1 h. Subsequently, the absorbance was measured at a wavelength of 517 nm using a spectrophotometer (Ocean Optics HR4000, Dunedin, FL, USA). The blank solution was prepared without the yogurt extract and the spectrophotometer was calibrated using ethanol [50]. The radical scavenging capacity of each yogurt sample was quantified as the percentage of the DPPH radical scavenging effect using the following formula:

(1) DPPH scavenging activity (%) = 100 × (1 − AS/A0),

where AS represents the absorbance in the presence of the yogurt sample and A0 represents the absorbance of the blank [51].

Total phenolic content evaluation: The yogurt samples’ total phenolic content was evaluated based on the Folin–Ciocalteu assay using a gallic acid calibration curve [47]. From the yogurt extract, 0.6 mL was combined with 3 mL of Folin–Ciocalteu reagent (0.2 N), and after 5 min, 2.4 mL Na_2_CO_3_ (0.7 N) solution was added. The resulting solution was incubated at room temperature (20 ± 2 °C) for 2 h, followed by measuring the absorbance at 765 nm using a spectrophotometer. The results were stated as μg of gallic acid equivalents (GAEs) per gram of yogurt [50].

### 2.6. Sensory Evaluation

The sensory analysis of the yogurt samples was performed by 15 trained panelists from the Food Engineering Faculty from “Ștefan cel Mare” University of Suceava according to the Romanian National Standard [52]. Appreciation of the color, aspect, and consistency was achieved by analyzing the samples in their packaging, and for the other characteristics (taste and flavor), the samples were distributed in glass beakers (30 mL). According to the standard, the consistency of the samples of acidic dairy products is appreciated at temperatures of 4–8 °C. The color was assessed immediately after opening the package in which the yogurt sample was kept. Each panel member assigned points from 0 to 5 to each sensory characteristic depending on the specific properties and deviations of the yogurt samples. Also, a coefficient of importance (weighting factor) was considered for aspect, color, consistency, and flavor (0.5), and for taste (2). The assigned points were multiplied with a weighting factor. On the basis of the total average score obtained, the quality level of the product was evaluated from an organoleptic point of view by classifying the quality levels from 0 to 20 points (inadequate to very good). The minimum quality condition, from an organoleptic point of view, for the delivery of milk or a dairy product is that it meets a total average score of at least 12.1 points.

### 2.7. Statistical Evaluation

All analyses were conducted in triplicate. For the sample’s differentiation, the analysis of variance ANOVA (α = 0.05) was conducted using Statgraphics Centurion XVI software version 16 and multiple comparisons of means using Fisher’s least significant difference (LSD) at the 0.95 confidence level. The Pearson correlation was performed with SPSS 16 (SPSS Inc., Chicago, IL, USA). Minitab version 17 (State College, PA, USA) was used for principal component analysis.

## 3. Results and Discussion

### 3.1. Physicochemical Characteristics of Yogurts Supplemented with Black Chokeberry

**Figure 3:**
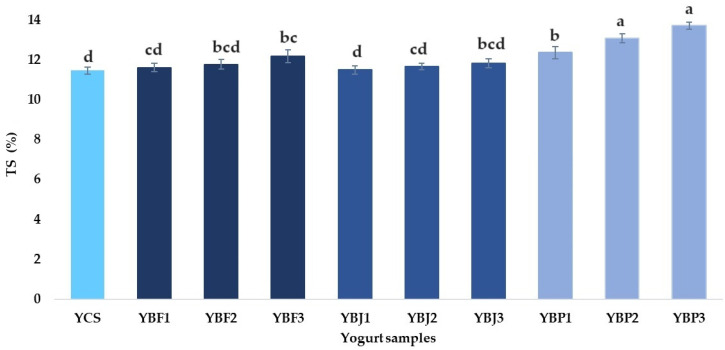
Total solids (TS) contents (%) of the yogurt samples. Means with different lowercase letters (a–d) indicate significant differences (*p* < 0.05) between the samples.

Total solids (TS) contents of the yogurt samples are illustrated in Figure 3 and ranged between 11.50 and 11.82% for the yogurt samples supplemented with black chokeberry juice, 11.61 and 12.18% for yogurt samples supplemented with black chokeberry fruit, and 12.36 and 13.71% for yogurt supplemented with black chokeberry pomace. The total solids content of the YCS sample was 11.46%, slightly lower than TS contents (12.82%) reported by Ibrahim et al. [53] and Dudal et al. [54] (12.4–12.8%), but close to 11.19% obtained by Alqahtani et al. [55]. According to Wang et al. [56], yogurt firmness depends on the total solids content of the mixture. The increase in the total solids content with the increase in the percentage of fruit pomace was also observed by Popescu et al. [57] and Varnaitė et al. [58].

**Figure 4:**
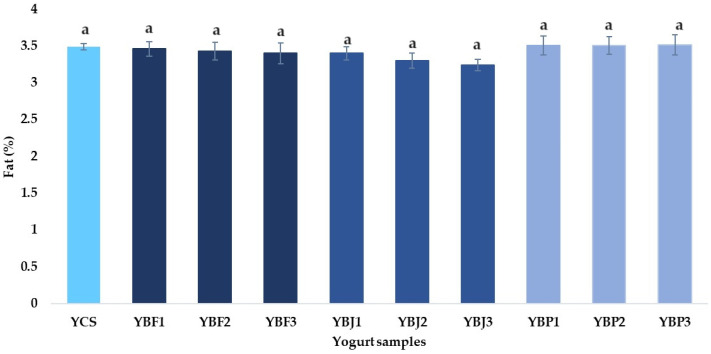
The fat content (%) of the yogurt samples. All means have a lowercase letter (a) indicating non-significant differences (*p* < 0.05) between the samples.

The control yogurt sample had a fat content of 3.49 ± 0.04%. A similar value (3.57 ± 0.11%) was reported by Plessas et al. [23]. The fat content of the yogurt samples supplemented with black chokeberry (Figure 4) varied from 3.24 ± 0.08% (yogurt sample with 3% black chokeberry juice—YBJ3) to 3.51 ± 0.14% (yogurt sample with 3% black chokeberry pomace—YBP3). It was observed that there are no significant differences between the samples, which means that the addition of black chokeberry does not result in a noticeable change in the fat content of the yogurt.

**Figure 5:**
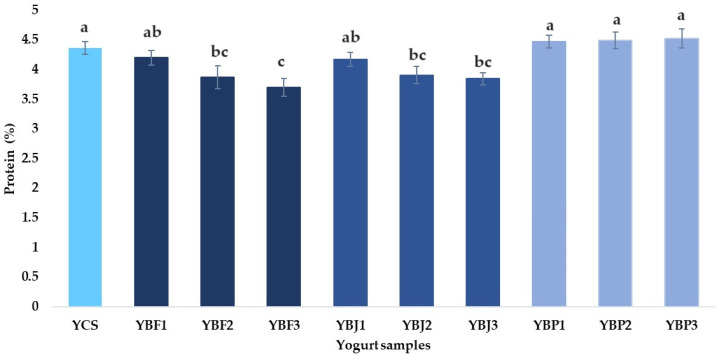
The protein content (%) of the yogurt samples. Means with different lowercase letters (a–c) indicate significant differences (*p* < 0.05) between the samples.

The protein content of the control yogurt (YCS) sample was 4.35 ± 0.11% (Figure 5).

Plessas et al. [23] obtained a protein content of 3.52 ± 0.05% for the control yogurt sample. In the present study, compared to the control sample, it was observed that the protein content decreased with the addition of black chokeberry fruit by 3.67% for the YBF1 sample, 11.03% for the YBF2 sample, and 15.17% for the YBF3 sample, while the addition of black chokeberry juice decreased the protein content by 4.13% for sample YBJ1, 10.34% for sample YBJ2, and 11.72% for sample YBJ3. According to Figure 5, the protein content of yogurt supplemented with black chokeberry pomace (between 4.46 ± 0.11% for YBP1 and 4.52 ± 0.16% for YBP3) was not significantly different from the protein content of the YCS sample.

**Figure 6:**
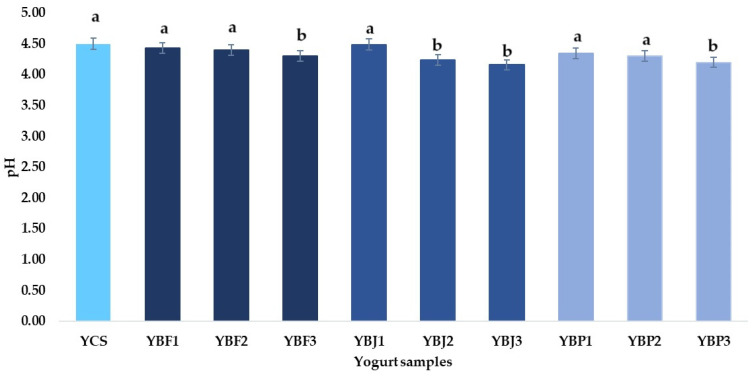
The pH of the samples. Means with different lowercase letters (a–b) indicate significant differences (*p* < 0.05) between the samples.

Lactose breaks down into lactic acid during milk fermentation, lowering the pH value [23]. The highest pH value was registered for the YCS sample (4.50), while the lowest was for the YBJ3 sample (4.16) (Figure 6). The pH values of the yogurt samples decreased due to the addition of fruit, juice, and pomace. Malic acid and citric acid are present in black chokeberry fruit, juice, and pomace, changing the pH value of yogurt. This was also observed by Du et al. [59] for stirred-type flavored yogurt with mulberry pomace (whose pH dropped to almost 4.30). In the present study, the pH values of the yogurt samples supplemented with 1, 2, and 3% black chokeberry pomace were 4.35, 4.30, and 4.20. Nguyen and Hwang [25] reported a pH of 4.29 for the yogurt samples with 1 and 2% chokeberry juice and 4.25 for yogurt with 3% juice, which is very close to those obtained for the YBJ2 and YBJ3 samples. All yogurt samples exceed the minimum of 0.6% lactic acid, which corresponds to yogurt according to [60]. The addition of chokeberry fruit, juice, and pomace increased the titratable acidity of the yogurt samples, possibly due to the presence of organic acids (such as quinic, malic, and citric acid [61]) in chokeberry, which can increase the acidity of yogurt [62]. According to King and Bolling [61], the TA of chokeberry fruit ranges from 0.85 to 1.22%, while Trenka et al. [63] determined TA values between 0.93 and 1.29% for chokeberry fruit from organic and conventional cultures. TA values between 0.89 and 1.06% as citric acid in chokeberry juices were reported by Tolić et al. [34]. Also, Tolić et al. [34] determined a TA (as citric acid) of 2.17% for chokeberry pomace powder, while Reißner et al. [64] obtained a TA value of 1.2% (expressed as tartaric acid equivalent). The results indicated that the TA of the yogurt samples supplemented with black chokeberry fruit (1–3%) ranged from 0.85 to 0.87%. Lower values (0.90%) were reported by Predescu et al. [24] for yogurt with 5% black chokeberry fruit. Nguyen and Hwang [25] obtained TA values above 1% (between 1.08 and 1.16%) for yogurt supplemented with chokeberry juice, which are slightly higher than those obtained in the present study. The TA values of yogurt supplemented with black chokeberry pomace were between 0.86 and 0.89%, falling within the values obtained by Demirkol and Tarakci [65] for grape pomace powder-fortified yogurt (0.78–0.93%).

The first sensory attribute observed by the consumers is color [22], which plays an important role in food product acceptance [66]. The color of yogurt was affected by the addition of black chokeberry fruit, juice, and pomace (Table 3). The L* values of the yogurt samples supplemented with 1% and 2% juice increased compared to the value of the control sample (YCS), while for the other samples, they decreased. Dimitrellou et al. [22] reported a value of 81.59 ± 1.87 for the color parameter L* for yogurt supplemented with black chokeberry juice. The addition of black chokeberry fruit to yogurt resulted in an increased a* value from −6.4 (YCS) to 3.9 (YBF1), 5.4 (YBF2), and 6.4 (YBF3), which means an increase in red color. The redness of the samples with black chokeberry fruit was more intense than the redness of the samples with black chokeberry pomace or juice. The yellowness of the yogurt samples decreased with the addition of black chokeberry fruit and pomace, while for the samples with 1 and 2% black chokeberry juice, the values slightly increased. Dark purplish fruit, black chokeberry, decreased L* and b* values, and increased a* values of the yogurt samples. This influence of black chokeberry on yogurt color was also observed by Nguyen and Hwang [25]. Different anthocyanin contents of yogurt samples can lead to significant differences in the color of the yogurt [67]. Yogurt color intensity and saturation are given by the chroma (C*) parameter and are calculated from a* and b* values [68]. C* values significantly decreased for the yogurt samples supplemented with black chokeberry fruit and pomace (Table 3). In the case of the sample, C* values decreased only for the sample with 3% juice addition (YBJ3), while for the other two samples, the C* values were not statistically different than the chroma value obtained for the control sample (YCS). The yellowness index (YI) represents the degree of yellowing by changing the color from white to yellow in the yogurt samples [69]. The YI decreased from 21.4 ± 0.1 (YCS) to 10 ± 0.1 (YBF2). It was observed that the YI decreased with increasing percentage of black chokeberry pomace in the yogurt samples. The lowest YI decrease was obtained for the yogurt samples supplemented with juice and the highest for the yogurt samples with black chokeberry fruit. The whiteness index (WI) increased with the increase in juice concentration in the yogurt samples (YBJ1 and YBJ2) compared to the control (YCS) and decreased with the addition of black chokeberry fruit and pomace. Hue angle (h°) decreased significantly with the increasing level of black chokeberry juice, fruit, and pomace. The largest decrease was observed for the yogurt with black chokeberry fruit samples, followed by the yogurt samples supplemented with pomace and juice. A hue angle of 90° indicates a yellow hue (maximum yellowness) of the samples [70]. Samples YBJ1, YBJ2, and YBJ3 were yellow–green with h° > 90, samples YBF1, YBF2 and YBF3 were more red–purple (h° value close to 0°), while the YBP1, YBP2, and YBP3 samples were more yellow-red.

The total color difference between the yogurt samples is presented in Table 4.

The difference in the yogurt control sample is visible to the human eye when the total color difference is greater than 3 [71]. The highest total color difference was observed for the yogurt samples supplemented with 2 and 3% black chokeberry pomace compared to the control sample, while the lowest total color difference (1.14) was obtained between YBF2 and YBF3, which means that the color of these two samples seems to be the same and only a few people will be able to detect the differences.

### 3.2. Texture Analysis of Yogurts Supplemented with Black Chokeberry

Hardness (as the force required to obtain a given deformation) is an important parameter for yogurt, according to Mousavi et al. [46]. Table 5 shows that the addition of black chokeberry fruit significantly increased the hardness of the yogurt samples, while no statistically significant changes were observed for the other yogurt samples with black chokeberry juice and pomace. The hardness of yogurt supplemented with black chokeberry ranged from 1.7 to 0.4 depending on the type of addition and its concentration. Mudgil et al. [72] indicated that adhesiveness is considered the force required to remove adhered material from the mouth while eating. Adhesiveness increased with increasing content of black chokeberry fruit and decreased with increasing percentage of black chokeberry juice and pomace added to the yogurt. Cohesiveness is related to the consumer acceptability of yogurt [72] and is defined as the forces of inner bond links [67]. The high cohesion values showed that the structure of yogurt from the samples containing black chokeberry is stronger and firmer compared to the control sample (Table 5). It was observed that cohesion increased with increasing amounts of black chokeberry fruit and juice and decreased with increasing amounts of black chokeberry pomace. Springiness is the rate at which the sample returns to its original dimensions after the deformation force is removed [46,72].

The textural integrity of yogurt is given by its elasticity (springiness); therefore, the addition of black chokeberry fruit increased the textural integrity of yogurt, while the addition of black chokeberry juice and pomace reduced it compared to the control sample (Table 5). It has been observed that as the percentage of pomace added to yogurt increases, the springiness of the yogurt decreases. Mousavi et al. [46] specified that gumminess is a defect and represents the energy needed to break down a semi-solid food until it is ready to swallow. The addition of black chokeberry fruit in yogurt enhanced gumminess compared to the control sample, while in the yogurt samples with 2 and 3% black chokeberry pomace, a decrease in gumminess was observed. Chewiness is related to firmness, cohesiveness, and springiness and represents the time required to chew a sample to reduce it to a ready-to-eat state [46]. Table 5 indicates that the addition of black chokeberry fruit to yogurt significantly increases the chewiness parameter, while the addition of black chokeberry juice and pomace significantly decreases it compared to the control sample.

### 3.3. Evaluation of the Antioxidant Activity of Yogurts Supplemented with Black Chokeberry

**Figure 7:**
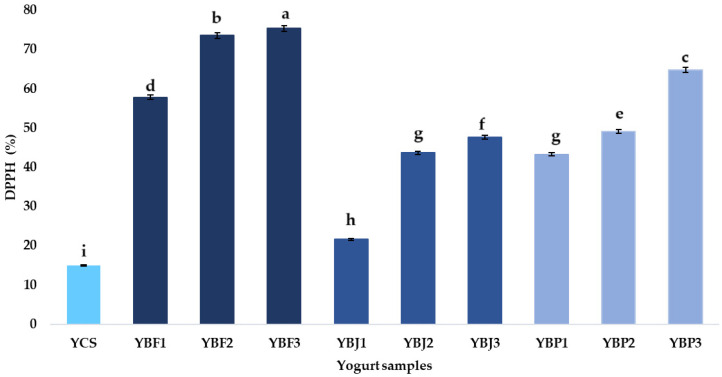
The DPPH (%) of the yogurt samples. Means with different lowercase letters (a–i) indicate significant differences (*p* < 0.05) between the samples.

The inhibition of DPPH (2,2-diphenyl-1-picrylhydrazyl) is one of the most commonly used assays for evaluation of the black chokeberry antioxidant activity according to Jurendić and Ščetar [73]. Olszowy-Tomczyk [74] indicated that higher antioxidant activity is provided by a higher percentage of inhibition. According to Krasnova et al. [75], the DPPH content in black chokeberry and their by-products (in TE mmol 100/g) is 13.09 ± 0.32 in fresh berries, 10.59 ± 0.45 in frozen berries, 21.16 ± 0.45 in pomace after juice pressing, and 2.85 ± 0.08 for juice from fresh berries. Adding black chokeberry and its by-products to yogurts can increase the antioxidant activity of yogurt. Figure 7 shows the antioxidant activity (DPPH) of the yogurt samples supplemented with chokeberry fruit, juice, and pomace. The DPPH radical scavenging activity of the control sample (YCS) was 15.02%, which is much lower than the 59.47% reported by Nguyen and Hwang [25] for plain yogurt. The use of fruit, juice, and pomace significantly influenced the DPPH values: the yogurt samples with chokeberry fruit presented the highest antioxidant capacity (up to 64.8 ± 0.11% inhibition), while the lowest was observed in the yogurt sample with 1% chokeberry juice (21.6 ± 0.11% inhibition). Marjanovic et al. [76] reported an antioxidant capacity for black chokeberry fruit of 87.68% inhibition, while Nguyen and Hwang [25] obtained an average inhibition of DPPH radical formation in yogurt containing 1%, 2%, and 3% chokeberry juice of 68.50%, 72.26%, and 77.87%, respectively. Petrov Ivanković et al. [77] indicated a 68.2% inhibition of DPPH radicals for chokeberry pomace. In the current study, it was observed that the inhibition of DPPH radical formation for 1% chokeberry fruit-supplemented yogurt was 2.68 times higher than that of 1% chokeberry juice-supplemented yogurt and 1.34 times higher than that of yogurt supplemented with 1% chokeberry pomace. In the case of the 3% chokeberry fruit-supplemented yogurt sample, the inhibition of DPPH radical formation was 1.58 times higher than that of 3% chokeberry juice-supplemented yogurt and 1.16 times higher than that of yogurt supplemented with 3% chokeberry pomace. These results indicate that black chokeberry fruit, pomace, and juice are advantageous options for food enrichment at the antioxidant activity level.

**Figure 8:**
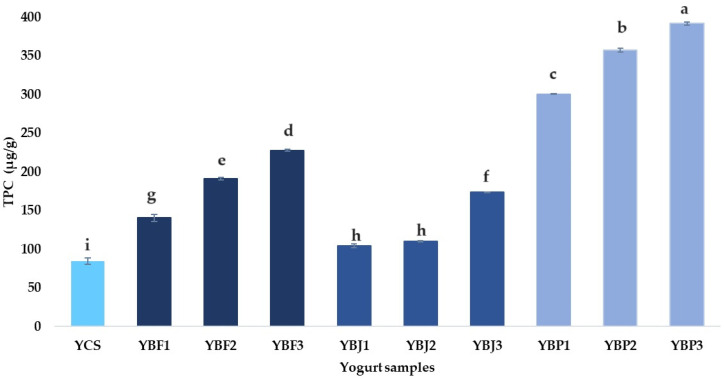
The total phenolic content (TPC, µg GAE/g) of the yogurt samples. Means with different lowercase letters (a–i) indicate significant differences (*p* < 0.05) between the samples.

Phenols may be found in cow’s milk due to cattle feed and/or aromatic amines could react with the Folin–Ciocalteu reagent resulting in a certain total phenolic content according to Okur [78]. Baniasadi et al. [79] reported a total phenolic content (TPC) of cow’s milk yogurt samples between 29.5 and 35.5 mg of GAEs per 100 mL, while Okur [78] indicated 20.25 mg of GAEs per 100 g of yogurt. In the present study, the total phenolic content was 8.48 mg of GAEs per 100 g of cow’s milk yogurt, which is much lower than the values found in the literature. In recent years, yogurt has been considered a delivery vehicle for phenolic compounds obtained from various sources (fruit, juice, pomace, and plant extracts) [80], as the low pH of yogurt contributes to the stability of phenolic compounds during storage [81]. Chokeberries’ strong antioxidant properties are provided by their high polyphenol content [73]. After the juice is extracted from the fruit, the phenolic compounds are retained in the chokeberry pomace [14]. Kapci et al. [82] reported 63 mg of GAE g DM^−1^ of total phenolic content (TPC), while Cacak-Pietrzak et al. [14] determined 54.04 mg of GAE g DM^−1^ of TPC in chokeberry pomace. From Figure 8, it can be observed that the TPC of yogurt significantly increased with the addition of chokeberry pomace, fruit, and juice. The higher content was registered for the yogurt sample with chokeberry pomace 3% (YBP3, 392.14 ± 2.06 µg GAE/g), while the lowest content was determined for the yogurt sample with 1% chokeberry juice (YBJ1, 104.45 ± 2.63 µg GAE/g). These values are higher than those obtained by Predescu et al. [24] for yogurt supplemented with 5% chokeberry fruit (41.56 ± 5.40 µg GAE/g) and by Dimitrellou et al. [22] for yogurt supplemented with chokeberry juice (56.5 ± 1.1 µg GAE/g). In the present study, it was observed that by adding 1% and 3% chokeberry fruit in yogurt, the TPC was approximately 1.66 and 2.68 higher than the control sample, respectively. A significant increase in TPC was observed in yogurts supplemented with chokeberry pomace with up to 4.62 higher content than for the control sample in the case of the addition of 3% pomace. In the case of yogurt supplemented with 3% chokeberry juice, the TPC was about 2.05 higher than the control sample. The relationships between the percentage of chokeberry fruit, juice, and pomace and TPC in the yogurt samples were linear (r = 0.987, *p* = 0.000; r = 0.811, *p* = 0.001; and r = 0.980, *p* = 0.000). Considering the obtained results, chokecherry (especially pomace) can be used as a source of phenolic compounds for the enrichment of dairy products, which are beneficial for human health.

### 3.4. Sensory Evaluation

**Figure 9:**
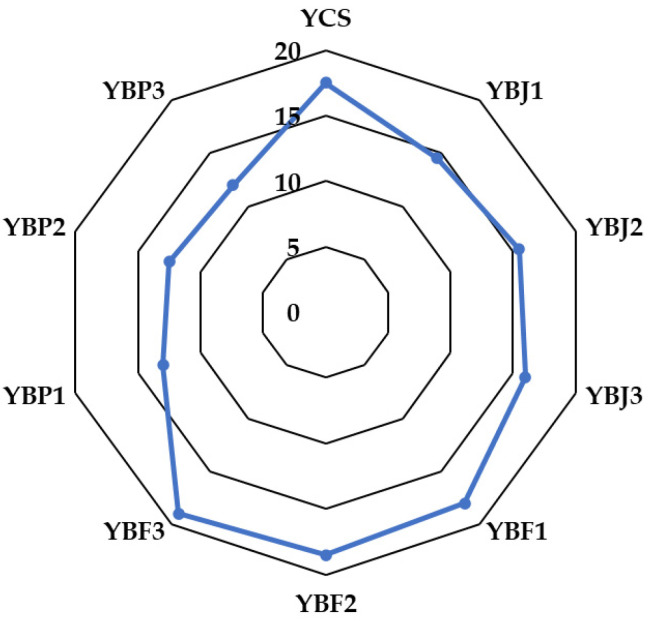
The sensory evaluation of the yogurt samples.

Ren and Chen [83] stated that 62–90% of the purchase decision is based on product color. In the present study, the color change was not very noticeable by evaluators in the yogurt samples with 1 and 2% black chokeberry juice added, while the color of all of the samples of yogurt with black chokeberry fruit was considered pleasant and was highly appreciated (receiving high scores according to sensory analysis). In the case of the samples supplemented with black chokeberry pomace, the color was more intense and darker and was not highly appreciated by the evaluators (probably because dark colors are associated with negative emotions like boredom and sadness [83]). The sensory evaluation of the yogurt samples is illustrated in Figure 9. The yogurt samples with the highest acceptance scores (19 and 18.5, respectively) were the samples supplemented with 3% and 2% black chokeberry fruit, while the lowest acceptance score (12) was determined for the yogurt sample supplemented with 3% black chokeberry pomace. The sample of yogurt with 3% chokeberry pomace had deficiencies or defects, indicating that it is inferior to the minimum quality of the product standard. Also, according to the score obtained, the samples of yogurt with 1 and 2% addition of chokecherry pomace presented weakly outlined specific properties, but also small defects, indicating that the product is at the minimum level allowed by the product standard. Decreasing the percentage of chokeberry pomace added to yogurt can lead to a better acceptance score.

### 3.5. Principal Component Analysis

**Figure 10:**
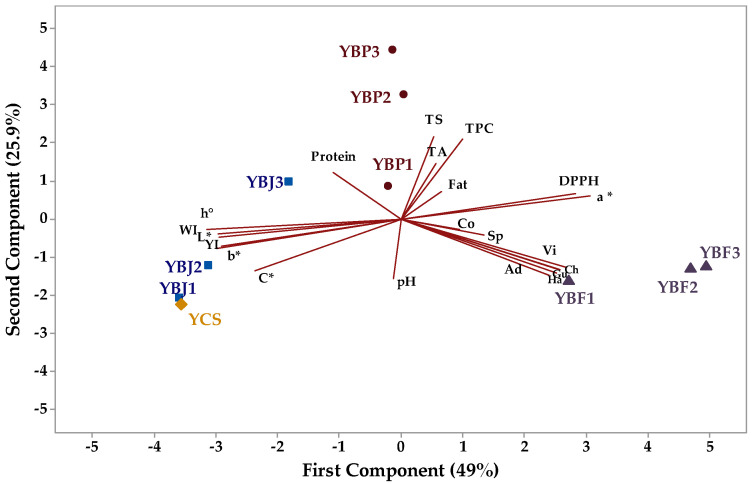
The principal component analysis biplot depicting the relationship between the parameters of the yogurt samples (TS—total solids, F—fat, P—protein, TA—titratable acidity, TPC—total phenolic content, Co—cohesiveness, Ch—chewiness, Ad—cohesiveness, Ha—hardness, Gu—gumminess, Sp—springiness, Vi—viscosity, YI—yellowness index, and WI—whiteness index).

The relationship between the physicochemical and textural parameters of the yogurt samples supplemented with chokeberry fruit, juice, and pomace is presented in Figure 10. The principal component (PC1) with an eigenvalue of 10.3 accounted for 49.0% of the total variation, while PC2 had an eigenvalue of 5.43 and accounted for 25.9% of the total variation. The negative loadings on PC1 were as follows: protein (−0.107), pH (−0.012), L* (−0.286), b* (−0.289), C* (−0.229), YI (−0.283), WI (−0.287), and h^0^ (−0.305). The other investigated parameters had positive loadings. TS (0.402), fat (0.132), protein (0.228), TA (0.268), DPPH (0.127), TPC (0.389), and a* (0.113) had positive loadings on PC2, while other investigated parameters had negative loadings. All yogurt with black chokeberry fruit (YBF) samples are situated to the right in the score biplot (Figure 10) and had positive values for PC1 and negative values for PC2. The control sample (YCS) and yogurt with black chokeberry juice (YBJ) samples are situated to the left in the score biplot, and the YCS, YBJ1, and YBJ2 samples had negative values for both PC1 and PC2, while YBJ3 and yogurt with black chokeberry pomace (YBP1 and YBP3) had negative values for PC1 and positive values for PC2. YBP2 had positive values for both PC1 and PC2.

## 4. Conclusions

Underutilized in the diet because of their acidity and astringency, chokeberry fruit, juice, and pomace may be natural sources of phenolic compounds for dairy products like yogurt. Adding chokeberry pomace to yogurt could also be a way of recovering pomace that may end up as waste. The formulation of new dairy products with different amounts of chokeberry fruit, juice, and pomace and the evaluation of their physicochemical, textural, and antioxidant characteristics can provide valuable information to fruit producers, dairy industry professionals, and consumers. To the best of our knowledge, until now, no studies have examined and compared all three chokeberry products (fruit, juice, and pomace) added to yogurt. The results obtained indicated that the total phenolic content was higher in the yogurt sample supplemented with chokeberry pomace and increased with increasing the percentage of pomace, which means strong antioxidant properties, while the yogurt samples with chokeberry fruit had the highest antioxidant capacity. Chokeberry fruit and pomace can act as a natural stabilizer and texturizing agent, improving the color, texture, and other qualities of yogurt. Consumers may choose such products because by adding chokeberry to dairy products such as yogurt, the astringent taste of chokeberry can be diminished. The effect of variable storage conditions on the shelf life of yogurt samples and microbiological analysis should be addressed in future studies.

## Figures and Tables

**Table 1:** The contents of components used in yogurt formulation.

Sample Coding	Main Sample Ingredients
YCS	Pasteurized cow milk, lactic cultures
YBF1	Pasteurized cow milk, lactic cultures, black chokeberry fruit (1%)
YBF2	Pasteurized cow milk, lactic cultures, black chokeberry fruit (2%)
YBF3	Pasteurized cow milk, lactic cultures, black chokeberry fruit (3%)
YBJ1	Pasteurized cow milk, lactic cultures, black chokeberry juice (1%)
YBJ2	Pasteurized cow milk, lactic cultures, black chokeberry juice (2%)
YBJ3	Pasteurized cow milk, lactic cultures, black chokeberry juice (3%)
YBP1	Pasteurized cow milk, lactic cultures, black chokeberry pomace (1%)
YBP2	Pasteurized cow milk, lactic cultures, black chokeberry pomace (2%)
YBP3	Pasteurized cow milk, lactic cultures, black chokeberry pomace (3%)

**Table 2:** The preparation of the yogurt samples.

Yogurt Samples	Cow Milk (%)	Black Chokeberry Fruit (%)	Black Chokeberry Juice (%)	Black Chokeberry Pomace (%)	Lactic Cultures (U)
YCS	100	-	-	-	20
YBF1	99	1	-	-	20
YBF2	98	2	-	-	20
YBF3	97	3	-	-	20
YBJ1	99	-	1	-	20
YBJ2	98	-	2	-	20
YBJ3	97	-	3	-	20
YBP1	99	-	-	1	20
YBP2	98	-	-	2	20
YBP3	97	-	-	3	20

**Table 3:** The ANOVA analysis of the yogurts’ color results.

Yogurt Samples	L*	a*	b*	C*	YI	WI	h°
YCS	74.6 ± 0.1 ^c^	−6.4 ± 0.1 ^j^	11.1 ± 0.1 ^b^	12.8 ± 0.1 ^a^	21.4 ± 0.1 ^a^	71.6 ± 0.03 ^c^	119.7 ± 0.1 ^a^
YBF1	55.2 ± 0.1 ^g^	3.9 ± 0.1 ^c^	4.8 ± 0.1 ^f^	6.2 ± 0.1 ^e^	12.4 ± 0.1 ^de^	54.8 ± 0.1 ^g^	50.8 ± 0.2 ^h^
YBF2	39. 8 ± 0. 2 ^i^	5.4 ± 0.1 ^b^	2.8 ± 0.1 ^g^	6.1 ± 0.1 ^e^	10 ± 0.1 ^f^	39.5 ± 0.1 ^i^	26.9 ± 0.2 ^i^
YBF3	40.2 ± 0.10 ^i^	6.4 ± 0.1 ^a^	3.2 ± 0.4 ^g^	7.2 ± 0.1 ^c^	11.6 ± 0.7 ^e^	39.7 ± 0.1 ^i^	26.8 ± 0.1 ^i^
YBJ1	87.6 ± 0.3 ^a^	−6.1 ± 0.1 ^i^	11.1 ± 0.6 ^b^	12.7 ± 0.5 ^a^	18.2 ± 1.0 ^b^	82.3 ± 0.6 ^a^	119 ± 1^b^
YBJ2	84 ± 1 ^b^	−4.7 ± 0.1 ^h^	12.2 ± 0.4 ^a^	13.1 ± 0.3 ^a^	20.8 ± 0.8 ^a^	79.1 ± 0.8 ^b^	111.2 ± 1. ^c^
YBJ3	65.1 ± 0.1 ^e^	−1.8 ± 0.1 ^g^	7.9 ± 0.1 ^c^	8.2 ± 0.1 ^b^	17.4 ± 0.1 ^b^	64.1 ± 0.2 ^e^	103 ± 0.1 ^d^
YBP1	69.5 ± 0.1 ^d^	0.2 ± 0.1 ^f^	6.8 ± 0.1 ^d^	6.8 ± 0.1 ^d^	13.9 ± 0.1 ^c^	68.70 ± 0.1 ^d^	88.34 ± 0.04 ^e^
YBP2	49.5 ± 0.1 ^h^	0.9 ± 0.1 ^e^	4.8 ± 0.1 ^f^	5 ± 0.1 ^f^	14 ± 0.1 ^c^	49.2 ± 0.1 ^h^	78.8 ± 0.1 ^f^
YBP3	61.9 ± 0.1 ^f^	3.6 ± 0.1 ^d^	5.4 ± 0.1 ^e^	6.2 ± 0.1 ^e^	12.6 ± 0.1 ^d^	61.4 ± 0.1 ^f^	60.70 ± 0.10 ^g^
*p*-value	*p* < 0.001	*p* < 0.001	*p* < 0.001	*p* < 0.001	*p* < 0.001	*p* < 0.001	*p* < 0.001
F-value	7324.36	28346.50	437.09	485.49	166.01	4274.91	8935.8

Different lowercase letters across columns (L*, a*, b*, C, h^0^, YI, and WI) denote a notable difference in average values (*p* < 0.05), as determined by one-way ANOVA analysis. L*—brightness, a*—red/green, b*—yellow/blue, C*—chroma, h^0^—tone, YI—yellowness index, and WI—whiteness index.

**Table 4:** The total color difference between the yogurt samples.

Yogurt Samples	YCS	YBJ1	YBJ2	YBJ3	YBP1	YBP2	YBP3	YBF1	YBF2	YBF3
YCS	-	12.99	9.35	10.96	9.35	26.88	16.73	22.76	37.67	37.52
YBJ1	-	-	4.20	23.10	19.66	39.26	27.82	34.43	49.86	49.64
YBJ2	-	-	-	19.34	16.04	35.51	24.12	30.68	46.08	45.84
YBJ3	-	-	-	-	4.96	16.17	6.34	11.83	26.84	26.66
YBP1	-	-	-	-	-	20.10	8.17	14.82	30.39	30.14
YBP2	-	-	-	-	-	-	12.64	6.48	10.87	10.89
YBP3	-	-	-	-	-	-	-	6.77	22.42	22.11
YBF1	-	-	-	-	-	-	-	-	15.66	15.35
YBF2	-	-	-	-	-	-	-	-	-	1.14
YBF3	-	-	-	-	-	-	-	-	-	-

**Table 5:** The textural properties of the yogurt samples.

Yogurt Samples	Hardness	Viscosity	Adhesiveness	Cohesiveness	Springiness	Gumminess	Chewiness
YCS	0.70 ± 0.2 ^b^	0.36 ± 0.03 ^d^	1.48 ± 0.3 ^bcd^	54.00 ± 0.1 ^f^	0.72 ± 0.02 ^c^	0.38 ± 0.01 ^e^	0.27 ± 0.01 ^cd^
YBF1	1.4 ± 0.3 ^a^	0.72 ± 0.05 ^b^	2.84 ± 0.1 ^a^	55.04 ± 0.1 ^e^	0.83 ± 0.03 ^ab^	0.77 ± 0.03 ^c^	0.64 ± 0.06 ^b^
YBF2	1.5 ± 0.2 ^a^	0.74 ± 0.01 ^b^	2.84 ± 0.2 ^a^	58.70 ± 0.3 ^c^	0.87 ± 0.04 ^a^	0.88 ± 0.05 ^b^	0.76 ± 0.02 ^a^
YBF3	1.7 ± 0.3 ^a^	0.84 ± 0.03 ^a^	2.97 ± 0.1 ^a^	59.23 ± 0.2 ^c^	0.75 ± 0.01 ^bc^	1.01 ± 0.02 ^a^	0.76 ± 0.03 ^a^
YBJ1	0.74 ± 0.2 ^b^	0.40 ± 0.01 ^cd^	1.73 ± 0.1 ^b^	52.28 ± 0.1 ^g^	0.57 ± 0.05 ^e^	0.39 ± 0.01 ^e^	0.26 ± 0.01 ^cd^
YBJ2	0.70 ± 0.2 ^b^	0.36 ± 0.02 ^d^	1.57 ± 0.2 ^bc^	52.34 ± 0.4 ^g^	0.66 ± 0.05 ^cde^	0.39 ± 0.00 ^e^	0.22 ± 0.05 ^de^
YBJ3	0.5 ± 0.4 ^b^	0.24 ± 0.01 ^e^	0.99 ± 0.1 ^de^	56.06 ± 0.2 ^d^	0.69 ± 0.01 ^cd^	0.25 ± 0.05 ^f^	0.17 ± 0.03 ^ef^
YBP1	0.8 ± 0.1 ^b^	0.44 ± 0.03 ^c^	1.86 ± 0.2 ^b^	64.32 ± 0.1 ^a^	0.61 ± 0.04 ^de^	0.50 ± 0.01 ^d^	0.31 ± 0.02 ^c^
YBP2	0.40 ± 0.3 ^b^	0.26 ± 0.01 ^e^	1.09 ± 0.2 ^cde^	62.86 ± 0.2 ^b^	0.55 ± 0.02 ^e^	0.23 ± 0.04 ^f^	0.17 ± 0.01 ^ef^
YBP3	0.4 ± 0.3 ^b^	0.24 ± 0.01 ^e^	0.88 ± 0.3 ^e^	60.31 ± 0.3 ^b^	0.41 ± 0.02 ^f^	0.17 ± 0.04 ^f^	0.09 ± 0.01 ^f^

Different lowercase letters across columns denote a notable difference in average values (*p* < 0.05), as determined by one-way ANOVA analysis.

## Data Availability

The original contributions presented in the study are included in the article, further inquiries can be directed to the corresponding author.
